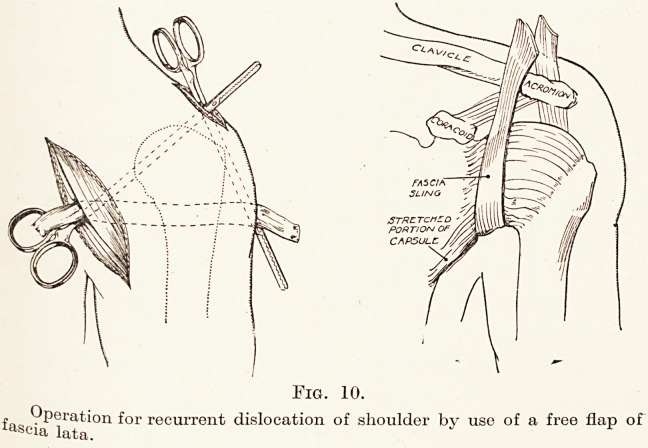# A Surgical Adventure: An Autobiographical Sketch

**Published:** 1933

**Authors:** Ernest W. Hey Groves

**Affiliations:** Consulting Surgeon, Bristol General Hospital; Emeritus Professor of Surgery in the University of Bristol


### The Bristol Medico-Chirurgical Journal

*"Scire est nescire, nisi id me*
*Scire alius sciret."*

SPRING, 1933.

### A SURGICAL ADVENTURE: AN AUTOBIOGRAPHICAL SKETCH.

The Presidential Address, delivered on 12th October, 1932, at the opening of the Sixtieth Session of the Bristol Medico-Chirurgical Society.

BY

Ernest W. Hey Groves, M.S., F.R.C.S.,
*Consulting Surgeon, Bristol General Hospital;*
*Emeritus Professor of Surgery in the University of Bristol.*

Every year a new man occupies the Presidential Chair, and every year he has to cudgel his brains to find new matter for a Presidential Address.

Many, in the endeavour to lead their hearers into new realms, have been tempted to plunge into unfathomable depths, or mount into the rarefied air of the stratosphere; but though I envy their audacity, I have neither the knowledge nor the courage to follow their example. I propose to stick strictly to earth, and to discuss some of the problems of medical practice as they have actually occurred in my own life.

Life must always be somewhat of an adventure, the mysterious reaction of the personality to his environment. This is particularly true of the medical profession, because the environment includes every kind of human being to be met, every kind of disease to be combated, and an ever-changing and increasing equipment of knowledge and experience with which to heal the sick bodies and the sad minds of our fellows. The many varieties of human beings and the manifold diseases have been the same since the earliest ages when the medicine-man set out on his adventure to heal his fellows, but the bounds of knowledge and the equipment of science have increased so much during the last century and last generation that the adventure has become more difficult and yet more interesting.

Perhaps a more modest form of address would be to attempt to review the progress of surgical science during the past thirty years. But then I could hardly avoid wearying you by the hackneyed nature of my subject. I am going to risk your condemnation for egotism in order that I may give a personal and intimate note to my remarks.

*The opening adventure.*—The first adventure of the profession is entering upon it. I suppose most men do this deliberately, after grave consultation with parents or teachers. It had been intended that I should go to India, the place of my birth, to follow my father's profession of engineering. It was the mere chance of seeing an advertisement of a scholarship at St. Bartholomew's Hospital, which included the exact subjects which I was taking in my Science Degree, which turned me into the path of the medical profession. I entered for this scholarship without consulting anybody—"just for a lark"—and when I

was successful it seemed fairly obvious that I ought to become a doctor.

My recollection of the five years of student life at Bart's is one of strenuous toil, rendered necessary by the fact that I had to earn my maintenance by teaching biology at the same time that I carried on my studies. I suppose it was this rather sordid struggle for existence which made me long for an independent position in which I might, to some extent, be my own master. At any rate, at that time I entertained no soaring ambitions, and only hastened to acquire the right to practise. Hence I was content to hold only one resident appointment, that of House Surgeon to the Obstetric and Gynæcological Department, working under Sir Francis Champneys, Dr. Griffith, and Mr. Harrison Cripps. For the same reason I was satisfied with the Conjoint Diploma, though fortunately in my routine work I had taken the Intermediate Examination of the London M.B., B.S., and the Primary Fellowship.

I can only recall one event of my student days which was at all adventurous. I had saved a little money, and as the result of a sudden impulse I went with a friend, F. A. Smith (now a Colonel in the I.M.S.), to Germany, as a student of Physiology at the University of Tübingen for one term. At the moment it seemed rather like waste of time and money, but I never regretted it in after years, because it gave me a groundwork in the German language, which has been invaluable to me since.

I am sure that it would be of great advantage to all students if for some period in their career they could go to a foreign University. It ought to be planned as part of the curriculum, and the time so spent counted as part of the present six years' course.

*The second step.*—Almost as important as the entry into the medical profession is embarking upon a career when legal qualification has been obtained. I am afraid I had only two guiding motives at this time: one was to earn my living, and the other to get into the country as the ideal contrast to the city life of London, which had been my lot for five and a half years. It required two years in the profound calm of Somerset air in Chewton Mendip to make me realize that I had only drifted into a backwater, and that it behoved me to get out once more into the active stream of life.

Country practice thirty years ago was a much more isolated existence than it is to-day. The radius covered by a horse or bicycle was not great, visitors were rare, and the only outdoor recreations those of riding a horse or planting a garden. Ample spare time enabled me to complete the M.B., B.S., and to obtain the M.D. degrees, although this was done much more as a pastime than with any ambitious idea of taking a higher status in the profession.

From the peaceful idleness of Chewton Mendip I went to that unkempt, industrial wilderness, Kingswood, where bootmakers, market gardeners, chapels and public houses struggle for existence. Certainly nothing seemed more unlikely than that such surroundings should give the opportunity and the incentive for entering upon a surgical career. Those were the days before National Health Insurance. The backbone of the practice consisted of a degrading form of contract work, known as "clubs," in which patients paid 4s. a year, children being half-price. The money was often collected by a tout, who himself drew a commission!

But there were two good things—plenty of work and interesting cases. It was the latter which, from a professional point of view, "saved my soul." Two of these cases stand out clearly in my mind as being among the first which brought me back into contact with the scientific medical world.

One was a patient with puerperal eclampsia. It occurred to me that the poison-laden circulation might be relieved by the transfusion of saline solution into the veins. This was carried out successfully in a poor cottage, with no assistance but that of a country midwife, and then I had the audacity to write up the case and urge this as a new method before such an august audience as that of the Obstetrical Society in London! But it was the beginning of that happy association with the leaders of the profession in Town which has continued and increased ever since.

The other case led to a much more daring adventure. It was that of a lady who had pain in the back, and who had been treated for more than a year by a physician in Clifton for "lumbago." She was bedridden, and from her couch used to watch the "new doctor," as I was then, go to his daily visit to his surgery at Hanham. Some feminine whim—or shall I call it intuition?—made her decide to call me in, and I found that she had an ovarian tumour impacted in her pelvis. She was so pleased at the discovery of a definite cause of her pain that nothing would satisfy her but that the doctor who had discovered it should also remove it!

For the moment this seemed to me unthinkable. I had no hospital or nursing home, or even the necessary instruments. But the lady insisted, and I was young and venturesome, and I felt that technically I was quite equal to the task. So, hastily, I improvised a room in my own house. My wife, who had been a nurse at Bart's, undertook to look after the case and the operation was successfully accomplished.

Naturally such a sudden leap from the sordid drudgery of club practice to the fascination of major surgery turned my thoughts definitely in the direction of operating work. As far as my Kingswood practice was concerned the new work added an untold zest to life, but it had a very dubious effect on my financial prospects. The fees paid were quite inadequate to compensate for the suspicions engendered in the minds of the public by my irregular and unusual conduct.

Gynæcological operations I had been accustomed to, of course, but the first time I ever took part in the removal of the appendix, the gall-bladder or prostate, or the performance of gastro-enterostomy was when I performed these operations in my own house, between the years 1898 and 1902.

Such a form of surgical practice cannot be too heartily condemned—in fact, it ought to be made illegal—but I can only plead in my self-defence that in those days all surgeons were doing these operations for the first time and that my irregular conduct was justified by success. I only had two deaths, one a case of tuberculous peritonitis and the other a pancreatitis complicating gall-stones.

I was necessarily brought into association with the consulting staff of the city in the course of my work, and I am greatly indebted to Dr. Freeman and to the late Dr. Michell Clarke, Mr. Richardson Cross, and Dr. Markham Skerritt, who advised me to write up my cases and present them to the Medico-Chirurgical

Society and the British Medical Association. By doing this I became known to the leaders of the profession, so that when an unexpected death vacancy occurred on the Staff of the General Hospital the unprecedented step was taken of appointing a general practitioner from the east end, who had had no previous association with that institution.

At the moment it seemed to me that the occurrence of the vacancy was just one of the " tides in the affairs of men, which taken at the flood, leads on to fortune," and I eagerly set out, little dreaming of the bitter struggle which lay before me in the years to come.

For four years I retained a share in the Kingswood practice, and used to toil out there daily on a pedal cycle for my club work, whilst in the afternoon I was the Consulting Surgeon in charge of out-patients at the Hospital. After this my livelihood was eked out by the help of coaching and by taking resident patients and students, while holidays were provided by doing seaside *locums.* What spare time I had was occupied in getting the Final F.R.C.S. and the London M.S.

*Adventures with the pen.*—I suppose there is only one more responsible thing than coaching students, and that is examining them, and I have had more than my share of both tasks.

As the period in which I was coaching and that in which I was preparing for the Fellowship coincided, the idea gradually evolved itself in my mind of tabulating my notes, so that a student could quickly look up any subject and get the salient points needed for a written or *viva voce* examination. And this idea gave birth to the *Synopsis of Surgery*, a work which, I venture to think, is not unknown to some of my audience.

The first edition appeared in 1908, and it was with some justice criticized as a dreadful cram book. But I am consoled by thinking that it has been a help to many thousands of students—and I have even heard complimentary remarks about it. The librarian at the University assures me it is the most stolen book which he stocks. I have just finished writing the tenth edition of the book, and whether it has been of service to anyone else, it has been of great value to me, because it gives me the opportunity every two years of revising the subject and of deciding how much of the old matter can go out and how much of new teaching is sufficiently established to go in. The quite unexpected success of this first adventure with the pen and my increasing contact with leaders of surgical thought set my mind at work on the subject of a surgical periodical.

The attempt to follow the current literature of the day made one conscious of the unpleasant fact that every leading country in Europe and America had its *Journal of Surgery*, whereas this country, the native land of Hunter and Lister, had nothing more than general medical journals. Inquiry about this strange fact showed that at least three times within the past generation an English surgical journal had been started and had then been given up. I was told that, apart from the financial backing of a wealthy society, the thing could not be made to pay, and that no publisher would undertake it; that surgeons would never send work to a new journal, lest such papers should be lost to the world by reason of the publication's speedy disappearance. However, with the incurable optimism of youth I went on, hoping that by a collective effort success might be achieved.

I was most fortunate in being in touch with Mr. Hartland Wright, of John Wright and Sons, who had published my *Synopsis*. Without his advice, encouragement and practical help the *British Journal of Surgery* would never have seen the light of day, or have survived to the mature age of which it now boasts.

Consideration of past failures in surgical journalism showed that these had been due to a want of wide responsible support by leading surgeons. We had to secure the active co-operation of one or more surgeons from every teaching centre in Great Britain, and we had to make sure that the active minds of the provinces should be joined to those in the Metropolis, so as to avoid the dangers of a publication in London, where the rivalry of the great schools has often prevented concerted effort. We were most fortunate in securing the active support and leadership of Sir Berkeley Moynihan and the co-operation of nearly all the progressive surgeons in every British teaching school. The first number appeared in July, 1913, and I can well remember the thrills of hope and fear that it caused us, and of the toil and anxiety necessitated by the effort to fill its pages with suitable material.

The Great War was the only serious threat to its survival, and in the end proved to be its salvation. In October, 1914, the Editorial Committee seriously debated whether it was worth while carrying on a surgical journal at that crisis in history. The war was to be over in three to six months, and then we could begin again. Fortunately, this short-sighted policy was not pursued, but, on the contrary, we organized special series of articles dealing with military surgery, and I think these articles established the reputation of the paper amongst not only British but also French and American surgeons.

Now that the *Journal* will be entering shortly upon its twenty-first year, we can feel fairly assured that it represents a real token and means of scientific surgical advance. The Editor's troubles are no longer concerned with the struggle to get enough material to fill its pages, but with the efforts to cope with the superabundance of material which he has at his disposal.

*The adventure with bones and joints.*— My early upbringing, with the idea of becoming an engineer, and a certain familiarity with tools and their use, gave me an inclination to those problems of skeletal repair and re-construction which form the basis of orthopædic surgery. The controversy aroused by Lane's work on the operative treatment of fractures in 1910 set me to work on this subject, and for some years I carried out experimental investigations on the repair of fractures under different methods of treatment. In those days (1910–1914) there were no facilities for experimental work on animals in Bristol, and I had to go up to London once or twice a week to the laboratories of University College. The toil and expense of this work were well worth while, for in a small way it gave me an education in exact methods which nothing could replace. The practical outcome of this investigation was to establish in my own mind certain facts which are now generally accepted. The chief of these are: the value of indirect fixation of bones by methods of skeletal traction (Fig. 1), the tendency of plating to produce either chronic osteitis (Fig. 2), or non-union (Fig. 3); that the essential conditions for bone grafting were wide and accurate contact of the graft with the host bone, and firm fixation, even more than the live autogenous nature

### PLATE I.

FIG. 1.

Tibia of cat twenty-one days after fracture, showing perfect position and good union treated by double transfixion.

FIG. 2.

Femur of cat six weeks after plating, showing proliferative osteitis.

FIG. 3.

Tibia of cat six weeks after plating, showing non-union.

of the graft. As a practical illustration of the application of this last-named principle I would take a striking example :—

A boy of 12 had a spontaneous fracture of his right humerus (Fig. 4, A) from throwing a cricket ball. The bone had a cystic cavity in the interior of its shaft. I excised this part of the bone (B) and replaced it by a double beef bone graft (Fig. 4, C), which took ample bearing on each part of the humerus by being driven deeply into its medullary cavity.

He was able to return to school in six weeks and made natural and healthy growth. The bone has grown of the same size and length as its fellow, and now, ten years after the operation, although a faint outline of the beef bone can be seen, it is obviously incorporated with the human bone in intimate vital connection. (Fig. 4, D.) Curiously enough, the little boy has grown into rather a gigantic athlete of 6 ft. 4 in., who this year at Cambridge won the competition for "putting the weight" (distance of 43 ft. $0\frac{1}{2}$ in.), which he actually did with the arm partly composed of beef bone.

It was just after I had finished the experimental research on bone injuries, and when the *British Journal of Surgery* was fairly launched on its second year of life, that the Great War came, and all our lives had to be re-adjusted to meet the new conditions. In two ways the war gave me a great impetus to pursue my work on the surgery of bones and joints. First, there was the very great wealth of material with which to work. Secondly, the organization of orthopædic work under Sir Robert Jones not only brought me under the sway of his gentle but compelling genius, but also associated me with other men doing this special work, both here and in France and America. My service with the Army took me for one year to Egypt, where I was in charge of the Surgical Division of the 21st General Hospital, and subsequently, in this country, I had surgical charge of one of the Military Orthopædic Centres at Fishponds, then at

Southmead, and later, after the Armistice, of the Pensions Hospital at Bath.

Apart from the horrors and tragedy of the war, those were gloriously happy years. The *camaraderie* of the profession was wonderful—not only did English, American, French, and Colonial surgeons get to know one another, but what was very much more strange,

FIG. 4.

Cystic disease of humerus treated by resection and grafting with beef bone. (A) Cystic humerus. (B) Portion of shaft resected. (C) Bone reconstructed with double beef bone graft. Tracing of X-ray fourteen days after operation. (D) Ten years later the X-rays show a faint trace of the graft, which has been almost wholly replaced by human bone.

the Surgical Staffs of the Royal Infirmary, General Hospital, and Royal United Hospital, Bath, worked together as a most friendly team.

Alas! those ideals, like others, such as "a world fit for heroes to live in" and "a war to end war," have tended to vanish in the post-war atmosphere, but their memory and influence remain, and one institution, the Bath and Bristol Surgical Club, survives to remind us that there still exists a local bond of union.

But to return to more strictly "Surgical Adventures." If it will not weary you I should like to describe some of the new things, both appliances and operations, which have been evolved from my work during those years of stress and in the quieter times which followed.

It was the problem of the gunshot fracture which produced the cradle splint. We were in Egypt with nothing but regulation equipment when the hundreds of the wounded were sent to us from Gallipoli. At first it was merely the idea of making a suspended rest, in which the wounded leg could be dressed at frequent intervals, which engaged our attention. We had the run of the engineer's stores and made a frame something like a Hodgen's splint out of iron rods a quarter of an inch thick, and fixed this to uprights. Then the necessity for allowing for traction became evident, and the uprights with pulleys were added. These cradle splints and all sorts of other surgical appliances were made in great numbers at a special factory which we equipped in Alexandria, native workmen being employed at the job. When the war was over, and we had to deal with pensioners and peace problems, it seemed likely that the various modifications of Thomas's splints and Balkan beams, which had been so largely used in France, would render the cradle splint superfluous. But I think that this has not been the case, and we are justified in continuing to use this splint, which has undergone some modification. A single pattern has taken the place of the three original types. The upper part is cut away so as to give room for the bed-pan, and two double sets of pulley wheels are provided so as to give a multiplied traction in one of two different directions. The value of the appliance is that it is a self-contained piece of apparatus, which provides for efficient traction treatment of the worst type of leg fracture, and yet is sufficiently light and compact to be easily taken about. Two other little improvements have helped this ideal of efficiency and compactness. First, the use of multiplying pulleys, two attached to the leg and two to the splint, by which the traction weight can be increased up to fourfold; and the second, a neat 10 lb. weight made like a large bobbin, which serves to wind up all the slack cord and which can be made fast at any point. (Fig. 5.)

Perhaps the most difficult fracture to deal with, either for the surgeon or the nurse, is that involving the spine. And it was the terrible problem of treating the gunshot wounds of the spine which set me thinking how it would be possible to fix a spinal case and yet provide for turning, inspection or dressing. At first we worked away with the high frames of the Pearson's bed, making a wooden frame to be suspended in this and revolved. This primitive pattern has gradually been superseded by others, and it is only during the last year that I have got a model which satisfies me. The patient lies in a frame which is concave from side to side. It is covered by a mattress which is pierced by a hole for the bed-pan. When he has to be

PLATE II.

Fig. 5.

Cradle splint for traction fractures of femur or leg bones.

In the model shown traction is applied by a transfixion pin. The weight is a 10-lb. bobbin on which the slack cord can be wound. This weight can be multiplied, two, three or four fold by the double pulley blocks.

turned he is covered by a second mattress, which contains a hole for his face, and then he is caged in by two hinged halves of a covering frame. When

FIG. 6.

Revolving spinal bed. In upper figure the frame is open. In the lower it is closed, and in this position the patient can be turned over.

these are fastened the patient lies securely held in a box six feet long, which is elliptical in transverse section. The whole of this cage can now be rotated so that he lies face downwards, and the back part of the frame, which is also hinged, can be undone and the patient's back exposed. (Fig. 6.) Thus, whether the spine is fractured or whether a bone grafting operation has been done, we have achieved a method by which perfect immobilization can be attained, and yet in which accessibility and exposure of the back can be assured, and this by a nurse working single-handed.

As I have already said, one of the most fascinating problems of reconstructive surgery is the possibility of transplanting human tissues so as to take the place of parts of the skeletal tissues lost by injury or disease. Skin and bone are the two tissues most often transplanted, but owing largely to the pioneer work of Gallie, the value of fascia for reconstructive work has been demonstrated. By the use of long strips of fascia lata the ligaments of the joints can be replaced or supplemented.

First, let me deal with the hips and the knee, where the ilio-tibial band can be used as a pedicle graft, allowing one end to remain in its natural attachment.

In the hip-joint paralysis of the gluteal muscle makes walking without crutches impossible, because the pelvis cannot be balanced on the leg, but simply drops down. If in these cases the tensor fasciæ femoris is still active, then the ilio-tibial band, which is really its tendon, can be freed from its lower attachment, brought up and drawn through the base of the great trochanter and attached to the superficial part of the erector spinæ muscle. By this means a strong abductor sling, formed of a new digastric muscle, is provided to take the place of the lost gluteals. (Fig. 7.)

In the knee-joint the crucial ligaments, and especially the anterior crucial, are frequently injured and torn across by any severe wrench which partly or wholly dislocates the knee. In those cases when the severity of the injury is not recognized at the time the knee is left in a very unstable condition. When the patient puts weight on the leg the tibia slips forward, giving the patient a great sense of insecurity, even if he is not thrown down. No

FIG. 7.

New muscle-fascia sling for paralysis of the gluteals.

A Tensor fasciæ femoris.
A[1] Fascia lata.
B Great trochanter.
C Head of femur.
D Erector spinæ.

apparatus will control this liability to dislocation unless it is cumbersome or locks the joint. It was when I was working at the Beaufort War Hospital that the problem first presented itself to me, and I was able to devise an operation which, with certain modifications, has now become standardized. The knee is opened from the front and the condition of the crucial ligaments verified. A long strip of fascia lata is isolated, leaving its lower attachment to the outer tuberosity of the tibia intact. This new ligament is then brought first through the outer condyle of the femur and then through the head of the tibia, so as to follow the original line of the anterior crucial. (Fig. 8.)

The same powerful, natural suture material is also available for repairs of the broken knee-cap. All surgeons agree that the usual transverse fracture of the patella needs to be fixed by open operation, because the fragments become drawn apart by the pull of the quadriceps, and because non-union is caused by the interposition of the flap of the aponeurosis. Wire is generally used, either as vertical sutures, holding the superficial parts of the bone, or else as an encircling band. But the drawback to this method of metallic suturing is that though it gives a strong union, yet it generally causes a bony overgrowth round the

(1.) — Condition of knee-joint on exposure at operation.

(2.)—Anterior crucial ligament replace[illegible] by band of fascia lata from muscl[illegible] covering thigh 12 inches long, 2 inche[illegible] wide. Fascia threaded through fem[illegible] and tibia by tunnels drilled in bone, an[illegible] finally fixed to tibia by ivory nail.

FIG. 8.

sutures, which leads to a condition like osteo-arthritis. If instead of encircling the bone by metallic wire a piece of the fascia lata is taken this bony excess is avoided. A strip of fascia is isolated, but with its lower attachment intact. It is threaded round the patella going through the quadriceps above and the ligamentum patellæ below. This not only makes a firm, strong suture of the broken bone, but the original attachment of the ilio-tibial band to the tibia gives a fresh, direct attachment of the extensors of the knee to the shin-bone. (Fig. 9.) In the shoulder-joint a new and powerful ligament can be made from a free piece of fascia lata. This method is of great value in the treatment of recurrent dislocation of the shoulder. After the joint has been dislocated several times a large gap is formed in the lower part of the capsule through which the humeral head is liable to slip whenever the arm is abducted and raised. A great number of different operations have been tried to remedy this condition, but it is very difficult to expose the real seat of the trouble, which lies deep in the axilla, and most of the operations suggested have led to failure. It seemed to me that what was required was a fascial sling as strong as the capsule itself, by which the head and neck could be prevented from slipping downwards and forwards.

In actual practice this operation has proved both simple and effective. A strip of fascia lata $1\frac{1}{2}$ inches wide and 8 inches long is prepared. Three incisions, each $1\frac{1}{2}$ inches long, are made, one in front, one behind, and one above the shoulder-joint. By blunt dissection with the point of the finger a tunnel is made right round the joint beneath the deltoid muscle and close to the capsule. The new fascial graft is passed from before backwards beneath the joint, and its two ends are brought upwards so as to meet and cross above the acromion, where they are fixed by silkworm-gut sutures. (Fig. 10.)

Perhaps — I fear probably — my account of my

FIG. 9.
Repair of fractured patella by means of an encircling band of fascia lata.

surgical adventures so far will have left the impression of a record of triumphs and successes, a blowing of trumpets, which I had better have left for someone else to do. But let me make a last effort to regain some semblance of modesty by the confession that my greatest adventure has been my greatest failure. It was in the years 1910 to 1914, just about the time of the conception and birth of the *British Journal of Surgery,* that I began regular visiting of the hospitals in this country and abroad with a travelling surgical club,

FIG. 10.

Operation for recurrent dislocation of shoulder by use of a free flap of fascia lata.

with the result that the ideas of reform of our hospital system dawned on my mind. Then followed the fateful years of the war, when the possibility and the advantages of organized co-operative work was demonstrated.

It was proved, for example, in connection with the gunshot fractures of the femur that the mortality was reduced from 80 per cent. to 10 per cent., not by the discovery of any new method of treatment, but chiefly by administrative efficiency, the chief elements of which were segregation and team work.

After the war was over it seemed so obvious to apply those lessons to the conduct of medical affairs in times of peace. The clear call in this city was for the two great voluntary hospitals to sink their differences, to join in one effective unit to which other and smaller hospitals would soon have been added. Then, and then only, would it have been possible to have organized special departments, such as orthopædics and a fracture service, worthy of a modern city; then, and then only, could clinical teaching units have been provided fit for a modern University; and then, and then only, would it have been possible to meet and join on fitting terms with the other great municipal hospital system of the city.

But these things were not to be, and we have to find what consolation we can from the spectacle of new hospitals springing up whilst the old hospitals struggle for a precarious existence. The University and the municipality, although friendly to the idea, seem powerless to initiate or to carry out any re-organization, and have to be content with an indefinite partnership with a hospital organization which they are impotent to control.

One almost fears that nothing short of some social cataclysm, such as Communism, bankruptcy, or war, will be strong enough to break down old prejudices, and to substitute a striving for the common good for the struggle for individual existence in the organization of our hospital service and the arrangement of clinical teaching.

*Acknowledgments are due to* Messrs. JOHN WRIGHT & SONS LTD. *for the loan of the blocks for Figs.* 1, 2, 3, 7, 10; *to* Messrs. CASSELL & CO. LTD. *for Fig.* 9; *to* Messrs. J. NESBIT, EVANS & CO. *for Fig.* 6; *to* "THE LANCET" *for Fig.* 4.

## Figures and Tables

**Fig. 1. f1:**
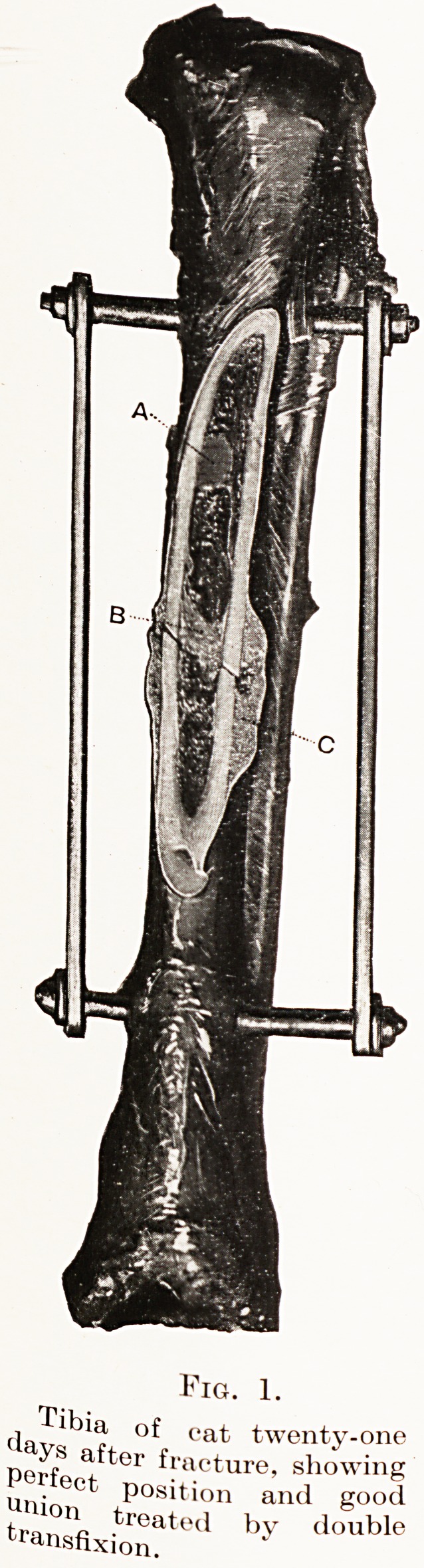


**Fig. 2. f2:**
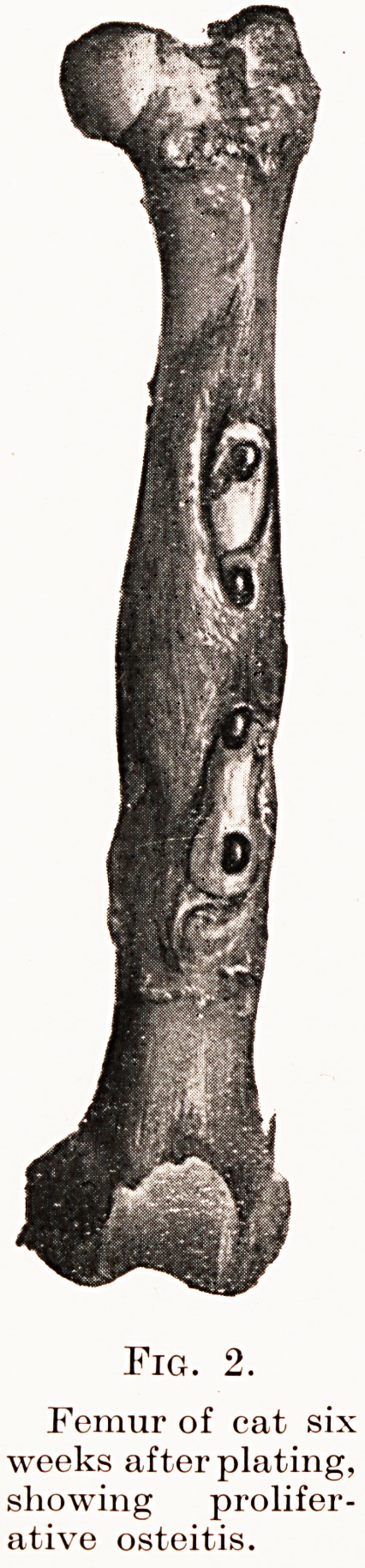


**Fig. 3. f3:**
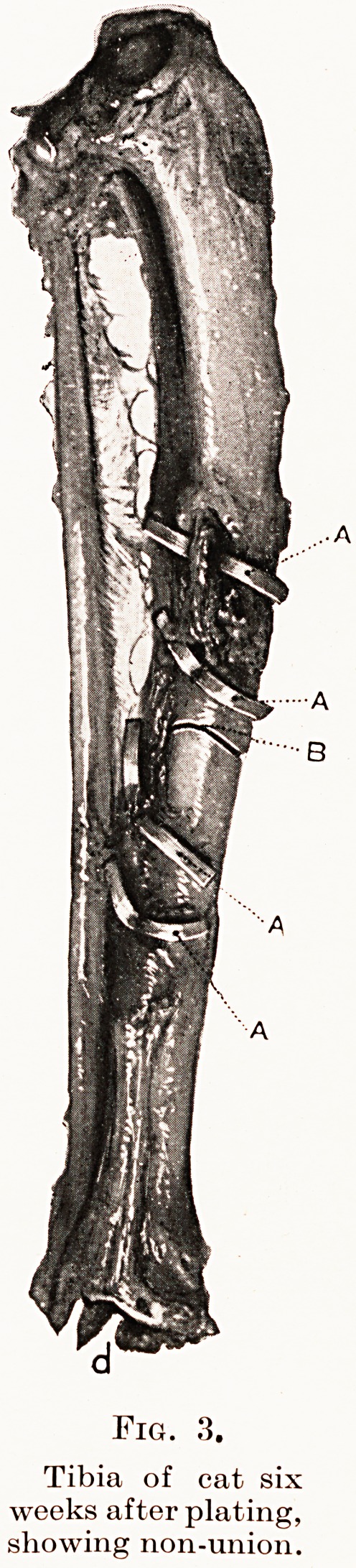


**Fig. 4. f4:**
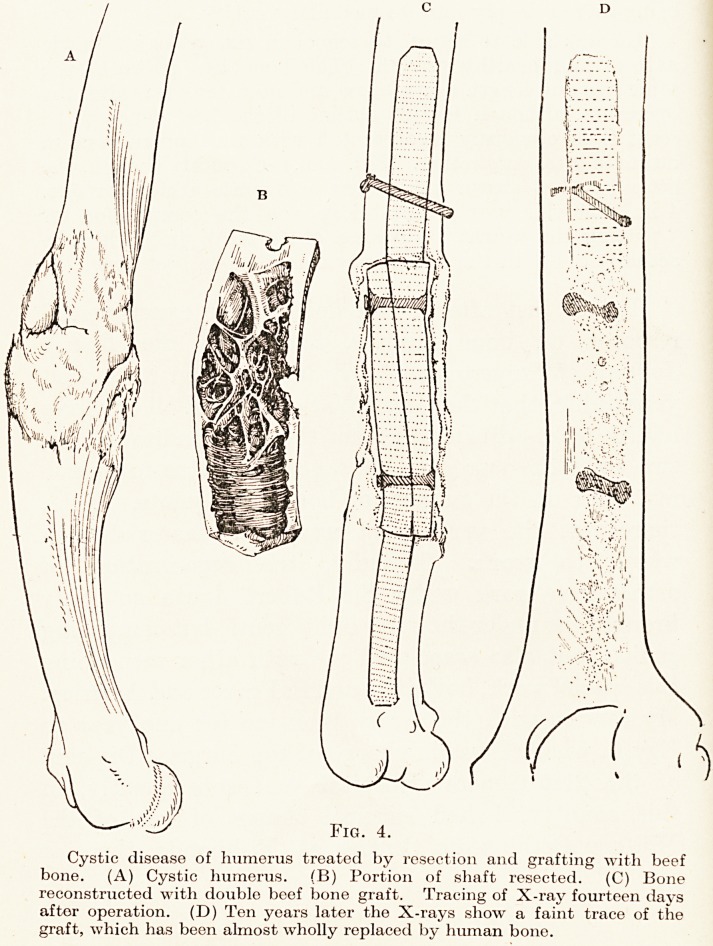


**Fig. 5. f5:**
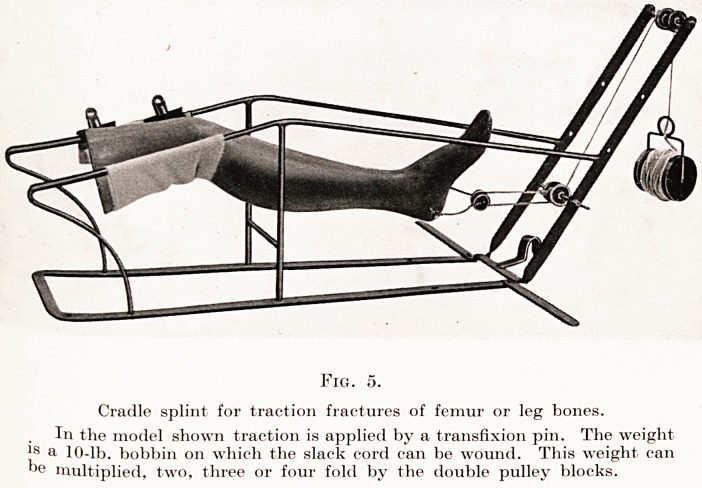


**Fig. 6. f6:**
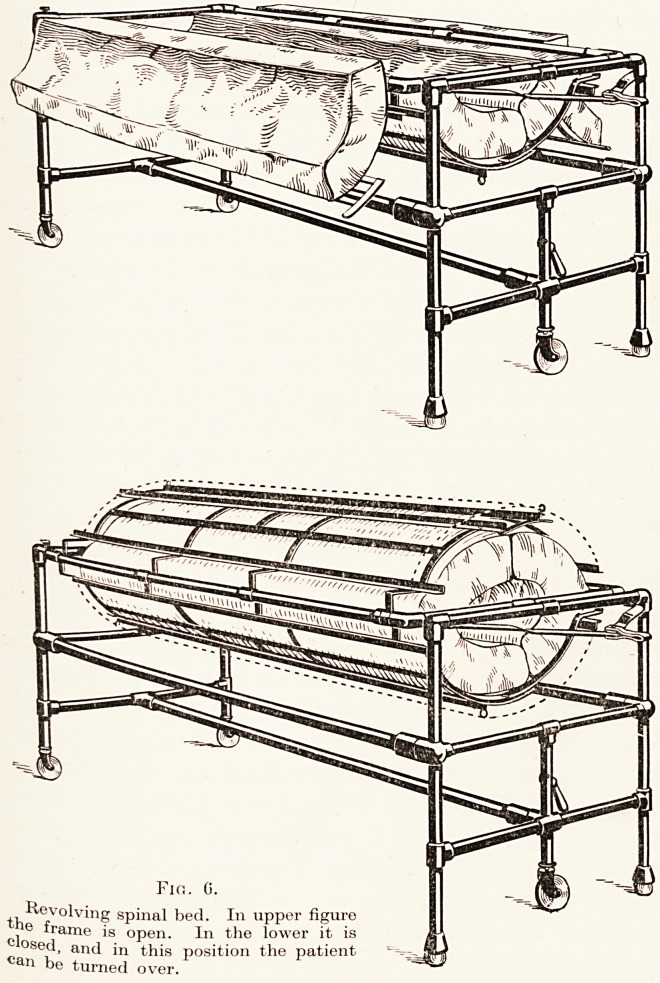


**Fig. 7. f7:**
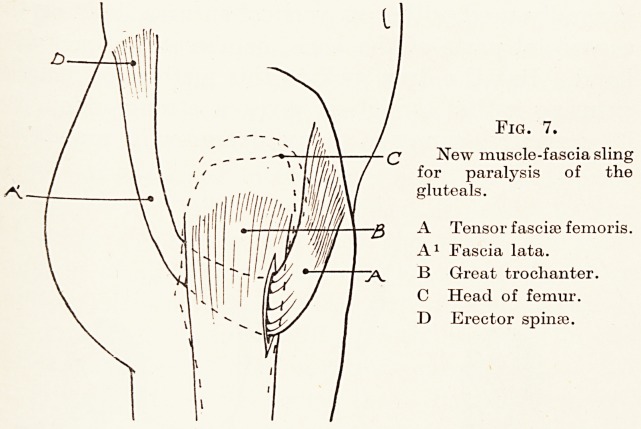


**Fig. 8. f8:**
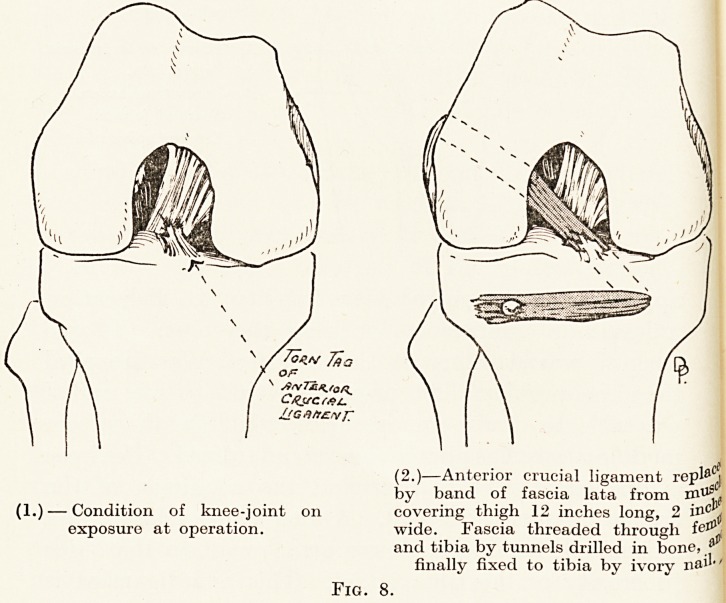


**Fig. 9. f9:**
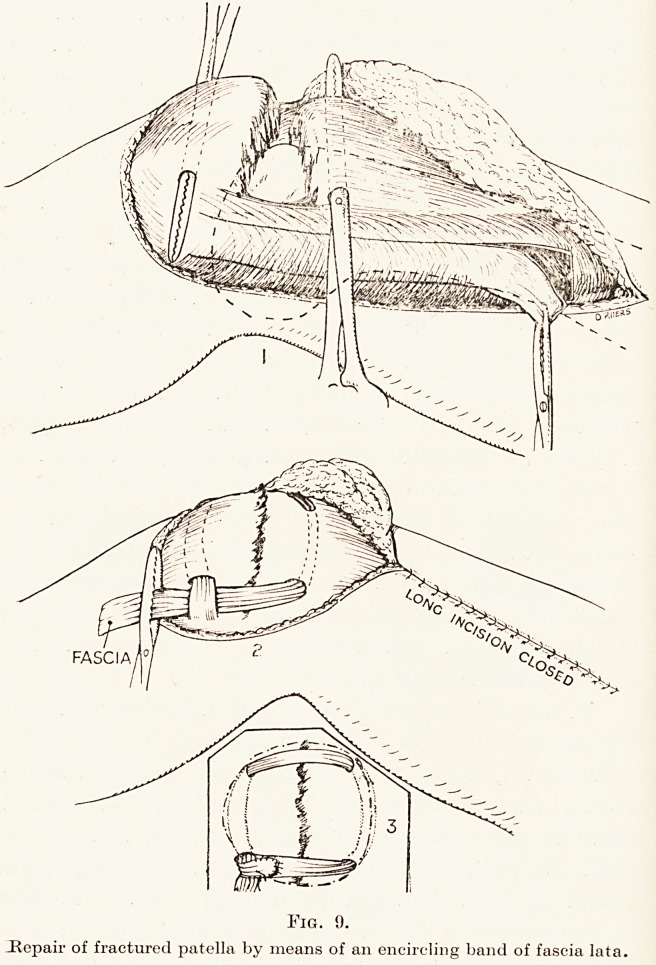


**Fig. 10. f10:**